# A Cell Line Resource Derived from Honey Bee (*Apis mellifera*) Embryonic Tissues

**DOI:** 10.1371/journal.pone.0069831

**Published:** 2013-07-23

**Authors:** Michael J. Goblirsch, Marla S. Spivak, Timothy J. Kurtti

**Affiliations:** Department of Entomology, University of Minnesota, St. Paul, Minnesota, United States of America; Goethe University Frankfurt, Germany

## Abstract

A major hindrance to the study of honey bee pathogens or the effects of pesticides and nutritional deficiencies is the lack of controlled *in vitro* culture systems comprised of honey bee cells. Such systems are important to determine the impact of these stress factors on the developmental and cell biology of honey bees. We have developed a method incorporating established insect cell culture techniques that supports sustained growth of honey bee cells *in vitro*. We used honey bee eggs mid to late in their embryogenesis to establish primary cultures, as these eggs contain cells that are progressively dividing. Primary cultures were initiated in modified Leibovitz’s L15 medium and incubated at 32^°^C. Serial transfer of material from several primary cultures was maintained and has led to the isolation of young cell lines. A cell line (AmE-711) has been established that is composed mainly of fibroblast-type cells that form an adherent monolayer. Most cells in the line are diploid (2n = 32) and have the *Apis mellifera* karyotype as revealed by Giemsa stain. The partial sequence for the mitochondrial-encoded cytochrome c oxidase subunit I (*Cox 1*) gene in the cell line is identical to those from honey bee tissues and a consensus sequence for *A. mellifera*. The population doubling time is approximately 4 days. Importantly, the cell line is continuously subcultured every 10–14 days when split at a 1:3 ratio and is cryopreserved in liquid nitrogen. The cell culture system we have developed has potential application for studies aimed at honey bee development, genetics, pathogenesis, transgenesis, and toxicology.

## Introduction

Established insect cell lines and primary culture methods are numerous and frequently used for diverse research interests such as understanding the transmission and pathogenesis of disease causing microbes. For example, one motivation for the establishment of the first continuous cell line from an insect, which was isolated from ovarian tissues of the emperor moth, *Antheraea* *eucalypti* [1], was to propagate viruses axenically for the purpose of developing control measures for agricultural and forest pests [2]. In the fifty years since this line was established, there have been over 500 continuous (i.e., immortalized) insect lines that have been developed, the vast majority (~80%) of which are dipteran or lepidopteran [3]. Moreover, advances in baculovirus expression systems used for recombinant protein production has made insect cell lines effective substrates for commercial and research applications [4]. Underrepresented, however, in the catalogue of insect lines are those derived from the order Hymenoptera (i.e., bees, wasps, and ants). Continuous cell lines from the hymenopteran lineage have been reported from only 6 species, including the pine sawfly *Neodiprion* *lecontei* [5] and the parasitoid wasps *Trichogramma* *pretiosum* [6], *T* *. confusum* , *T* *. exiguum* [7], *Mormoniella* *vitripennis* [8], and *Hyposoterdidymator* [9].

Despite the economic and ecological importance of honey bees as pollinators of many cultivated and native plants, there is a surprising lack in availability of controlled *in vitro* systems, especially given that several threats to honey bee health are obligate intracellular pathogens that are abundant and widespread across colonies [10]. A limited number of studies have documented attempts at culturing honey bee embryonic cells [11–13] and larval and pupal cells [14–19]. Short-term cultures (≤ 4 weeks) have been demonstrated with neurons dissociated from honey bee pupal antennal lobes [17,20]. Long-term cultures have been initiated using pre-gastrula embryos (36–40 h after oviposition) that remained mitotically active for 3 months [12]. The limited duration of cell survival and absence of lines gave rise to the tenet that honey bee cells were refractory to continuous *in vitro* growth. Difficulty in adapting honey bees cells to *in vitro* conditions may be the result of selecting donor tissues whose age or origin is unsupportive of long-term growth. Recently, gene transfer technology has been used to evade these limitations, where insertion of the green fluorescent protein gene by lentivirus transduction [13] and the human *c-myc* proto-oncogene by lipofection [21] into embryonic honey bee cells was performed to demonstrate if activation of the transgenes was feasible and could promote long-term proliferation and survival. The latter method resulted in the establishment of a cell line that remained viable during an 8-month follow-up period; however, subsequent evidence to support claims of a continuous line has not been forthcoming. Our objective was to use standard insect cell culture techniques without the use of retroviruses or transfection of human oncogenes to isolate honey bee cell lines. Herein, we report the isolation and characterization of a cell line, which we have named AmE-711, from primary cell cultures derived from fragmented honey bee embryonic tissues. At the time this manuscript was submitted, the AmE-711 line has been passaged 18 times and remains in culture.

## Materials and Methods

### Ethics statement

No specific permits were required for the described field studies. Observations were conducted at the University of Minnesota apiary; therefore, no specific permissions were required for this location. The apiary is the property of the University of Minnesota and not privately-owned or protected in any way. Field studies involved observing the European honey bee (*Apis mellifera* L.), which is neither an endangered or protected species. The honey bee cell line reported below is an original description of a line that was developed by the authors at the University of Minnesota from honey bee embryos. The cell line was isolated from an insect; no institutional review board or ethics committee approval was needed.

### Mass collection of honey bee eggs

A honey bee colony was visually inspected for the absence of signs of brood diseases (i.e., American foulbrood, European foulbrood, and Chalkbrood) before it was selected for the collection of eggs. An empty frame of drawn-out comb was placed in the center of a selected brood box within the colony for 24 h to allow the worker bees to clean the comb cells in preparation for the queen to lay eggs. After 24 h, the queen from the colony was restricted to one side of the empty frame for 24 h using a metal cage that covered the entire side of the frame. Queen-attending nurse bees were small enough to pass freely between the wire mesh of the cage. After being restricted for 24 h, the cage was removed and the queen released. The frame was examined for the presence of eggs and subsequently returned to the colony for incubation. Between 48 and 72 h after oviposition (i.e., after the release of the queen), the frame was removed from the colony and brought to the laboratory.

In the laboratory, the frame was struck at an acute angle several times against a tabletop covered with clean packing paper similar to the method of Evans et al. [22]. The side of the frame that contained the eggs faced down as it was struck, which allowed the eggs to fall onto the paper. Macroscopic debris, such as wax flakes, was removed and the eggs deposited into a sterile 15 mL conical tube. This method allowed for efficient collection of several dozen to hundreds of eggs per frame.

### Culture medium and supplements

The basal medium was Leibovitz’s L15 medium (Life Technologies, Grand Island, NY) [23] modified according to Munderloh and Kurtti [24]. Briefly, the modifications included the addition of glucose, organic acids, vitamins, trace minerals and amino acids to the L15 base. This basal medium has been used previously to culture insect and tick cell lines [24–26]. The complete medium (HB-1) for honey bee cells was prepared by mixing 3 parts of the basal medium with one part of cell culture grade distilled water and supplementing this with fetal bovine serum (FBS; 10%), tryptose phosphate broth (5%; BD Biosciences, Franklin Lakes, NJ), bovine lipoprotein-cholesterol concentrate (0.1%; MP Biomedical, Aurora, OH), HEPES (10 mM), and NaHCO_3_ (0.9 mM). The pH was adjusted to 7.0–7.2 with 1 N NaOH.

### Primary culture

Honey bee eggs were surface sterilized with sequential washes of 0.525% sodium hypochlorite containing Tween 80, 0.5% benzalkonium chloride, and 70% ethanol followed by several rinses with sterile water. The water was removed and ≤ 200 µL of HB-1 medium was added to the tube. The eggs were homogenized in medium by gently pressing a sterile 1.5 mL pestle (Kimble Chase, Vineland, NJ) against the eggs to disrupt the chorion, releasing the inner embryonic fragments into the medium. The homogenate was then transferred to a Nunclon flat-sided culture tube with an effective growth area of 5.5 cm^2^ and non-ventilating screw cap (Thermo Fisher Scientific, Inc., Waltham, MA) containing 500 µL medium with the addition of 100 U/mL penicillin, 100 µg/mL streptomycin, and 0.25 µg/mL amphotericin (Life Technologies, Grand Island, NY). The sides of the conical tube were washed 1 or 2 times with ≤ 500 µL medium to collect any residual embryonic fragments and then transferred to the culture tube. The screw cap was then tightened and the culture containing ≤ 2.0 mL total volume of medium was moved to a non-humidified incubator set at 32°C. Culture medium was replaced 1 or 2 times a week or when a sharp drop in pH was noted by a change in phenol red indicator.

### Subculture and preservation of the cell line

Transfer or passage was initiated when the monolayer of a primary culture (or subculture) was ≥ 80% confluent. At the time of transfer, the medium was removed and the cell layer was trypsinized (0.25% trypsin-EDTA; Life Technologies, Grand Island, NY) for several minutes at 32°C to dissociate the cell layer from the flask substrate. Trypsinization was stopped with fresh medium and the cell suspension was transferred at a split ratio of 1:2 or 1:3. However, a ratio as low as 1:10 allowed for continual expansion of the AmE-711 line but extended the interval until the next subculture.

Starting at the 3^rd^ transfer, cultures were selected for cryopreservation to assure retention of characteristics and a banked source of the subsequent line. Once cultures were ≥ 80% confluent, cells were dissociated as above. The cells were then pelleted by centrifugation at 400 g for 5 min at 4°C. The cells were resuspended in chilled freezing medium comprised of L15 base with 20% FBS and 10% dimethyl sulfoxide. The suspension was aliquoted into cryotubes and frozen at a rate of ^-^1°C per min using a Handi-freeze tray (Union Carbide, Houston, TX) or CoolCell alcohol-free freezing container (BioCision, LLC, Mill Valley, CA). Cryotubes were transferred to liquid nitrogen for long-term storage. Cells stored in liquid nitrogen were regenerated successfully. The AmE-711 line is currently cultured in HB-1 medium without antibiotics and has been screened and found negative for *Mycoplasma* sp. using a LookOut detection kit (Sigma-Aldrich, Co., St. Louis, MO) (Curt Nelson unpublished data) and by light microscopy of cells stained with Giemsa.

### Conventional PCR

We used PCR sequencing of amplicons to confirm the species identity of the AmE-711 line. Genomic DNA was extracted from the whole abdomen of a 20-day old adult honey bee, honey bee embryos, a larva of the Common Eastern bumblebee, *Bombus* *impatiens* , AmE-711 cells, and HL-60 human promyelocytic leukemia cells using a DNAeasy blood and tissue kit (Qiagen, Valencia, CA) according to the manufacturer’s recommendations. DNA yield was determined by spectrophotometry prior to PCR.

Each PCR reaction contained ~14–18 ng of template, 0.2 µM of previously published forward (5’-ttaagatccccaggatcatg-3’) and reverse (3’-gttatccacgtcataaacgt-5’) primers [27] specific for the amplification of *A. mellifera* mitochondrial-encoded cytochrome c oxidase subunit I gene (*Cox 1*), puReTaq Ready-to-Go beads (GE Healthcare, Piscataway, NJ), and 22 µL of nuclease free water. Reactions were run using a thermal profile consisting of an initial step of 94°C for 5 min, followed by 40 cycles of a 3-step protocol consisting of 94°C for 20 sec, 40°C for 1 min, and 72°C for 1 min, and a final step of 72°C for 5 min. Amplified products were resolved on a 1% agarose gel by electrophoresis, stained with a 1:5,000 dilution of Gel Green fluorescent nucleic acid stain (Biotium, Inc., Hayward, CA), and visualized with UV light.

Honey bee embryo and AmE-711 amplicons for *Cox 1* were purified by Diffinity Rapid Tip 1 purification tips (MidSci, St. Louis, MO) according to the manufacturer’s protocol. Purified products were checked by spectrophotometry and ~60 ng of template and 10 pM of forward-only or reverse-only primers used for PCR as above were diluted with nuclease free water and submitted to the University of Minnesota Biomedical Genomics Center for Sanger Classic automated sequencing. Sequences were manually aligned to remove primer artifacts and queried against the BLAST database. The partial *Cox 1* sequence for the AmE-711 line was deposited in Genbank (Entry: KC921208).

### Karyology

AmE-711 cells in log phase growth were exposed to 12.5 µM colchicine in fresh medium and incubated at 32°C for 24 h. After 24 h, the medium was removed and the cells were dissociated with 0.25% trypsin for < 10 min at 32°C. Dislodged cells were pelleted by centrifugation at 270 g for 8 min and then resuspended in 4 mL of 75 mM KCl and incubated at 34°C for 60 min. The cells were pelleted by centrifugation at 270 g for 8 min and resuspended after the addition of 3:1 methanol: acetic acid fixative. The cells were pelleted again and resuspended in fresh fixative and incubated at room temperature for 30 min. The cells were pelleted a third time and resuspended in 200 µL of fresh fixative. Fixed cells were dropped onto slides pre-chilled at ^-^20°C and allowed to air dry. Slides were stained with 3.2% Giemsa in Sorenson’s buffer, pH 6.8, for 60 min at 34°C. Chromosome spreads were photographed using a DXM 1200 image capture device attached to an Eclipse E400 phase contrast microscope with 100X oil objective (Nikon, Inc., Melville, NY). Chromosome counts were determined from 126 cells in metaphase arrest.

### Growth analysis

AmE-711 cells were dissociated as above and the suspension was used to inoculate three 12.5 cm^2^ culture flasks at a density of 3.0 x 10^5^ cells/mL. The flasks were placed in an incubator at 32°C and HB-1 medium was replaced every 3 days. Starting at 24 h after inoculation and every 24 h thereafter until the cultures reached confluence, the number of cells in 10 random fields from each of the 3 flasks was counted manually using phase contrast microscopy. Average cell density ± SD was plotted per cm^2^ on a linear scale against time (days).

### Effect of temperature on cell growth

AmE-711 cells were dissociated as above and the suspension used to inoculate 15 flat-sided culture tubes (5.5 cm^2^) with 2.25 x 10^5^ cells per tube. Tubes were randomly assigned to the baseline condition (i.e., harvest within 24 h at 32°C after inoculation) or one of four temperatures after inoculation: 25, 28, 32, and 34°C. HB-1 medium was replaced once during a 7-day incubation period. After 7 days, the medium was aspirated and the cell layer was washed twice with 1X DPBS (without Ca or Mg; Mediatech, Inc., Manassas, VA). Tubes were incubated overnight at room temperature with 0.5 mL of 0.5 N NaOH to solubilize proteins and stored at 4°C until analysis. 250 µL of Quick Start Bradford 1X dye (Bio-Rad, Hercules, CA) was added to 5 µL of solubilized protein to separate wells of a sterile flat bottom microtiter plate (Sarstedt, Inc., Newton, CA). Samples were thoroughly mixed and absorbance read at 595 nm using a VersaMax microplate reader (Molecular Devices, LLC, Sunnyvale, CA). Three replicate tubes were established for each treatment and 3 replicate wells were assayed from each tube. Data were normalized against a reagent blank and bovine serum albumin (BSA) standard set (Bio-Rad, Hercules, CA). Mean total protein per unit surface area of tube substrate (cm^2^) was analyzed by one-way ANOVA and post-hoc comparisons for temperature were carried out by Tukey’s HSD test (α = 0.05).

### Effect of commercial medium on cell growth

AmE-711 cells were dissociated as above and the suspension used to inoculate 18 flat-sided culture tubes with 2.0 x 10^5^ cells per tube. Tubes were incubated for 48 h at 32°C in HB-1 medium (baseline). After 48 h, the medium was aspirated and the cell layer was washed once with 1X DPBS. Tubes were then randomly assigned to receive complete medium containing a different commercially available base: Grace’s, ILP-41, L15 (i.e., HB-1), Schneider’s, or Shield’s. All media, except L15, were purchased from Sigma-Aldrich, Co. (St. Louis, MO). Tubes were incubated for an additional 5 days and processed for protein as above. Three replicate tubes were established for each treatment. Mean total protein was analyzed and post-hoc comparisons determined for commercial media as above.

## Results

### Features of primary cultures and young cell lines

Nearly 100 primary cultures were prepared from fragmented honey bee embryonic tissues with the method described (or with slight variation in procedures). Inoculum size varied, but as little as 25 embryos was sufficient to inoculate one 5.5 cm^2^ tube and initiate a primary culture. A 5.5 cm^2^ flat-sided culture tube with a non-ventilating cap was the preferred plasticware for establishing primary cultures as the relative surface area and volume were small and minimized evaporation.

**Figure 1:**
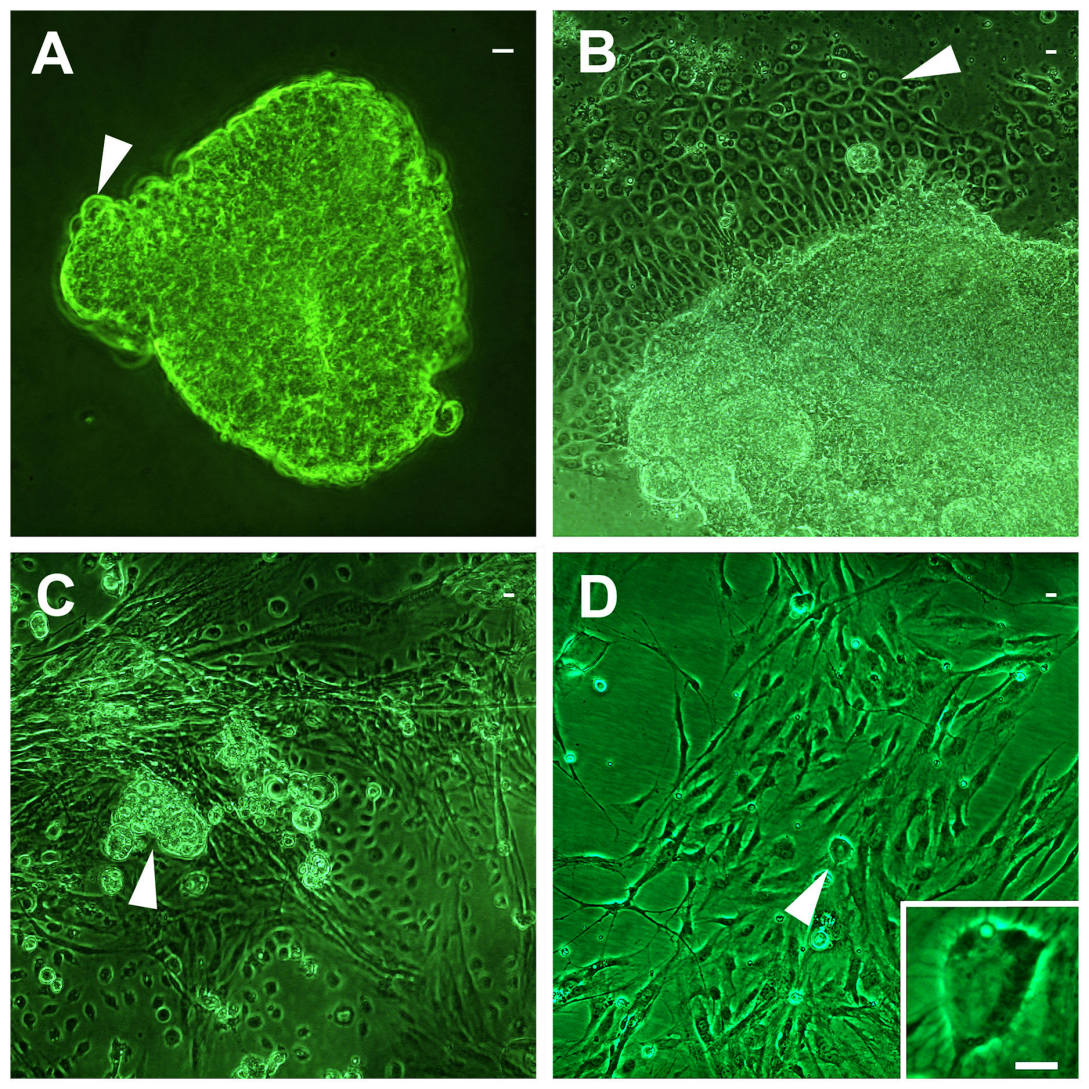
Phase contrast micrographs of honey bee cells. Cell characteristics depict different stages in the culture process: A. Round, undifferentiated cells (white arrowhead) of an unattached tissue fragment from a newly inoculated primary culture. **B**. Outgrowth of tightly packed epithelial-like cells (white arrowhead) from a tissue explant in an early stage primary culture. **C**. A remnant tissue explant (white arrowhead) in a primary culture at 1 month that is nearly confluent with heterotypic cell arrangements, most notably elongated fibroblast-type cells. **D**. A monolayer of mainly fibroblast-type cells that are loosely packed (~60% confluent) in the AmE-711 cell line that had been transferred 14 times. White arrowhead indicates a rounded, refractile cell undergoing mitosis, enlarged in the lower right corner inset. Scale bar = 10 µm.

Explants of embryonic tissue became loosely adherent within 24 h after inoculation. There was difficulty in distinguishing the morphology of the cells, and it was not possible to determine the origin of the rudimentary tissues, in these fragments. Cells in these explants were undifferentiated, appearing large and round similar to those described by Bergem et al. [12] (Figure 1A). However, cells that migrated away from the tissue explants were firmly attached and had a differentiated morphology, unlike Bergem et al. [12]. In the initial days of culture, an aggregation of squamous-shaped epithelial-like cells was often noted as the first distinguishable cell type that migrated away from, and formed a periphery around, the tissue explants. The formation resembled an epithelial sheet of tightly packed cells (Figure 1B). The epithelial-like cells had a high nuclear–cytoplasmic ratio and contained several prominent nucleoli. Within the first week to one month, most primary cultures showed cells that had migrated away from the tissue explants and were highly heterotypic (Figure 1C). However, fibroblast-type cells became the dominant cell type in most advanced cultures (Figure 1D). Fibroblast-type cells were spindle-shaped, had a densely staining nucleus by Giemsa, and a low nuclear–cytoplasmic ratio. In addition, thin, elongated cytoplasmic extensions of some fibrous cells in an advanced state of differentiation were capable of exhibiting undulating contractions.

### Isolation of the AmE-711 line

**Figure 2:**
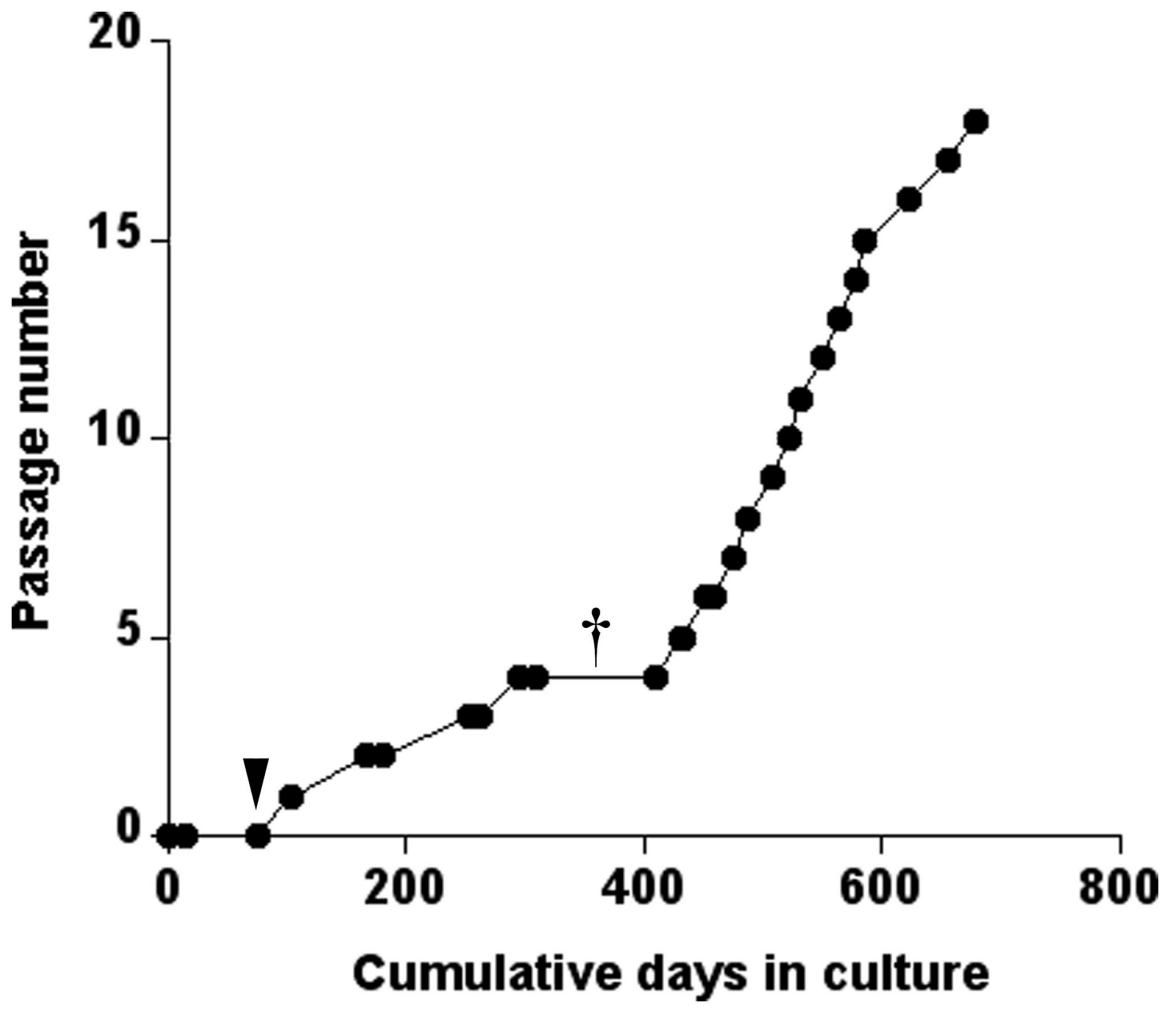
Passage number and cumulative days of AmE-711 cells in culture. The black arrowhead indicates the date when AmE8-11 (inoculation date: 7/8/11; culture day 0) and AmE9-11 (inoculation date 7/22/11; culture day 14) primary cultures were combined. Several additional primary cultures were isolated between 10/20/11 and 11/10/11, and non-adherent material (i.e., tissues fragments, single cells) collected within days after inoculation of these cultures was added to the combined AmE8-11 and AmE9-11 flask. The combination of these two primary cultures, along with material from other primary cultures, would later become the AmE-711 cell line. † indicates the lifespan of many primary cultures and young cell lines isolated using the method described.

Most of the primary cultures that we were able to subculture reached confluence within 3 months of inoculation. We were able to subculture approximately one-third of all primary cultures. After the first transfer, the frequency of subsequent transfers or passages was unpredictable and only one line (AmE-711) was taken past 5 transfers (Figure 2). Enzymatic dissociation by trypsin, as opposed to mechanical techniques such as sloughing, tapping the flask, or scraping, has been suggested as effective for dislodging strongly adherent insect cells from the flask substrate prior to transfer [28,29]. We also observed that trypsinization for < 10 min at 32°C allowed for a homogenous suspension to be obtained for re-seeding and that the cells to be transferred were insensitive to the proteolytic activity of trypsin exposure. However, incubation periods with trypsin > 10 min led to cells that were slow to re-attach and/or injured, likely through irreversible damage of protein function [30].

### Characterization of the AmE-711 line

### Karyotype

**Figure 3:**
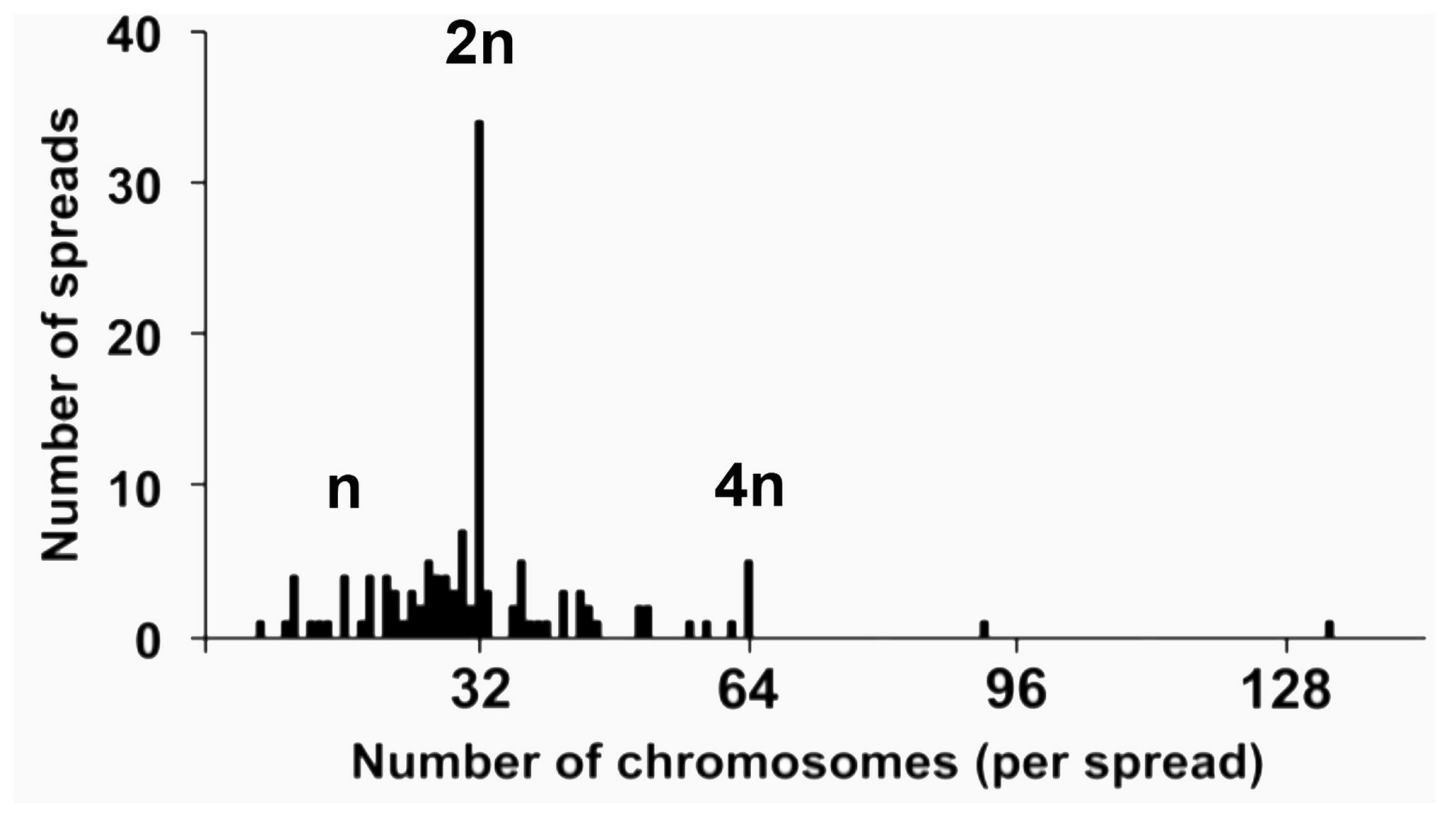
Distribution of chromosomes of AmE-711 cells. The AmE-711 line had been transferred 8 times. A total of 126 metaphase spreads were counted. Haploid, diploid, and tetraploid conditions are demarcated by n, 2n, and 4n, respectively. Chromosome counts greater than 64 (4n) were considered as polyploid.

**Figure 4:**
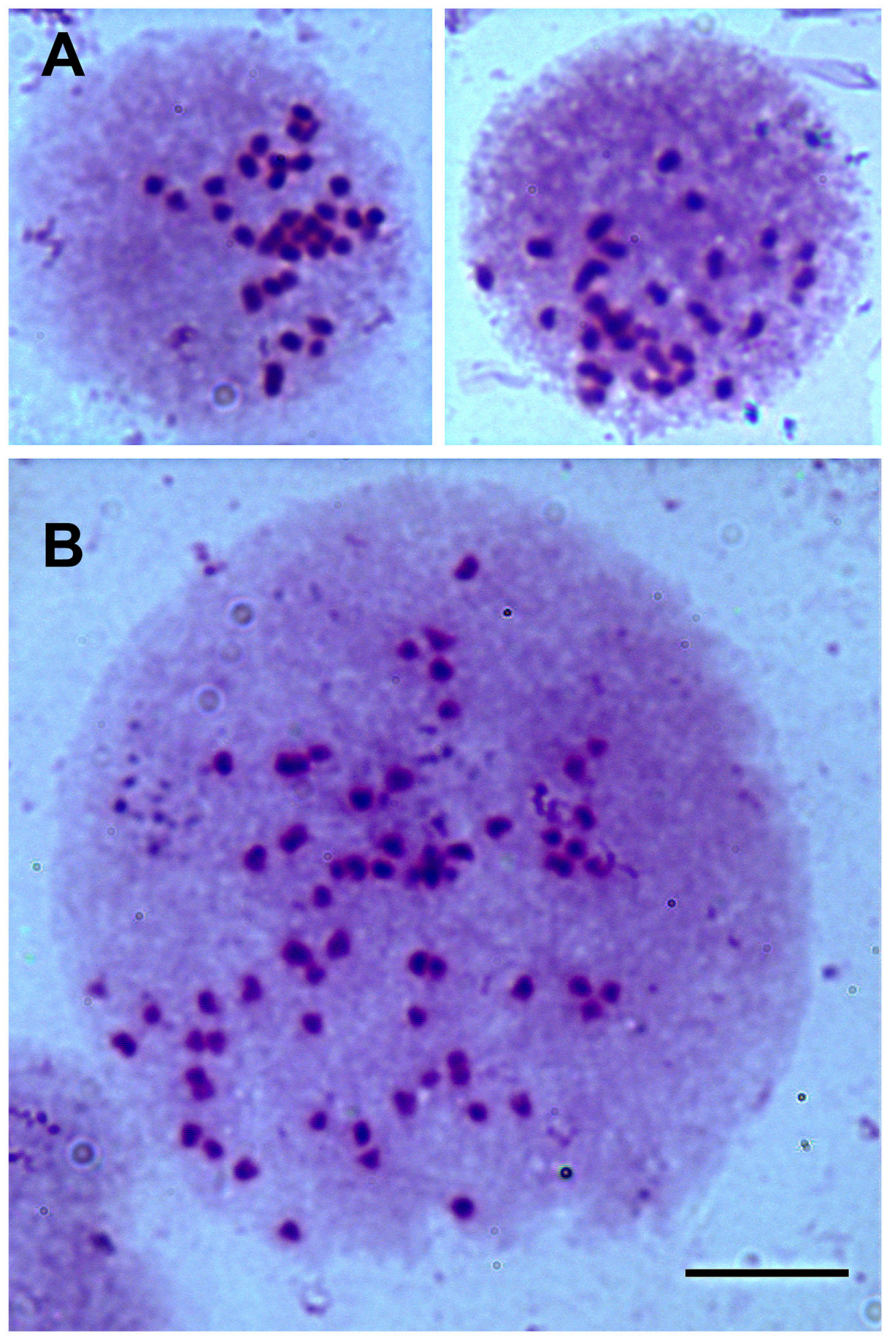
Chromosomes of the AmE-711 cell line. Chromosomes were prepared from a brief (40-min) incubation with 0.125 mM colchicine in hypotonic KCl solution. **A**. Two representatives of diploid (2n = 32) chromosome spreads in nuclear matrix. **B**. A tetraploid (4n = 64) chromosome spread in nuclear matrix. Scale bar = 10 µm.

The expected haploid and diploid karyotypes for *A. mellifera* are n = 16 and 2n = 32, respectively [31,32]. From 126 chromosome spreads, the range in chromosome number we observed was 6–133 (Figure 3). The modal chromosome number was 32, and 50% of the spreads had 26–36 chromosomes. Chromosomes were small (< 2 µm), making it difficult to identify individual chromosomes by diagnostic characters such as banding pattern and arm length. Some of the variability in chromosome number that we report can be explained, in part, to this small size, which could be resolved with higher magnification or other staining techniques. However, all cells in our culture system have a karyotype indicative of *A. mellifera* origin (Figure 4A). Polyploidy was rare and was observed in only two sets (< 2.0%) that had chromosome numbers of 92 and 133. Moreover, 3.2% and 4.0% of the sets we observed had a karyotype that was haploid or tetraploid (4n = 64) (Figure 4B), respectively.

### Species identification by polymerase chain reaction (PCR)

**Figure 5:**
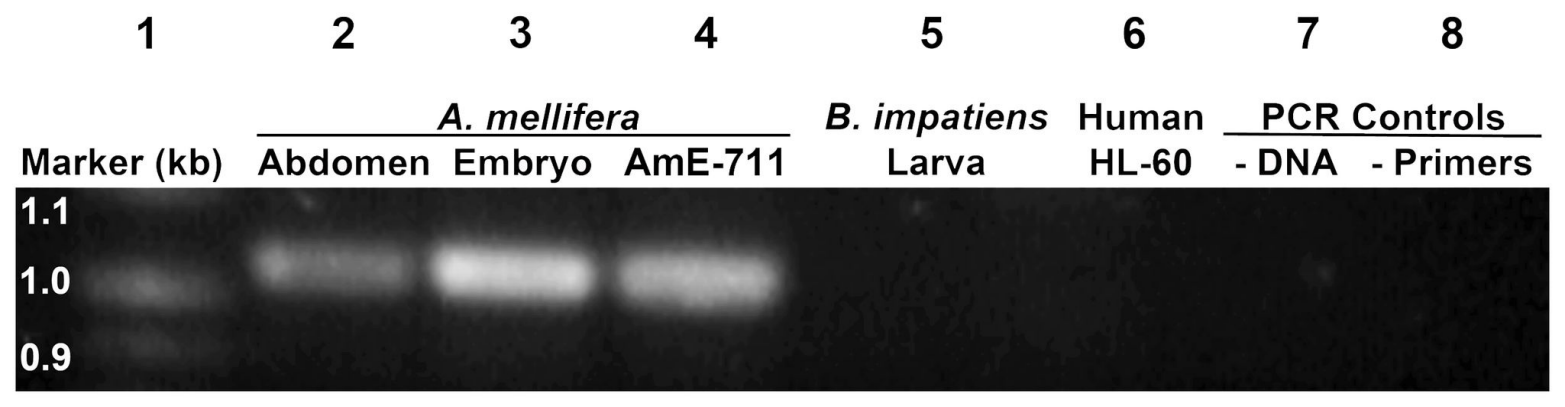
Approximate 1050-bp region within the mitochondrial-encoded cytochrome c oxidase subunit I gene (*Cox 1*) from samples of genomic DNA amplified by PCR. Lane 1, marker (100 bp); lane 2, *Apis mellifera* adult abdomen; lane 3, *A. mellifera* embryo; lane 4, AmE-711 cells; lane 5, *Bombus* *impatiens* larva; lane 6, HL-60 human promyelocytic leukemia cells; lane 7, Taq polymerase and primers without DNA; lane 8, Taq polymerase and AmE-711 DNA without primers.

PCR products amplified with primers specific for the *A. mellifera* Cox *1* gene showed a positive signal for DNA from adult honey bee abdomen, honey bee embryos, and AmE-711 cells (Figure 5). The amplicon matched closely the 1044 bp amplicon for *Cox 1* reported by Hall and Smith [27] and Corona et al. [33]. No signal was observed for the larva of the Common Eastern bumblebee, *B* *. impatiens* (negative control), HL-60 human promyelocytic leukemia cells (negative control), or PCR controls. Comparative sequence analysis showed that the amplicon for the AmE-711 line was identical to that of the honey bee embryo and to a 956 bp sequence of the complete *Cox 1* gene (sequence position, 416–1371; Genbank Entry: M23409.1) [34] and *A. melliferaligustica* mitochondrial genome (sequence position, 1952–2907; Genbank Entry: L06178.1) [35].

### Cell growth

**Figure 6:**
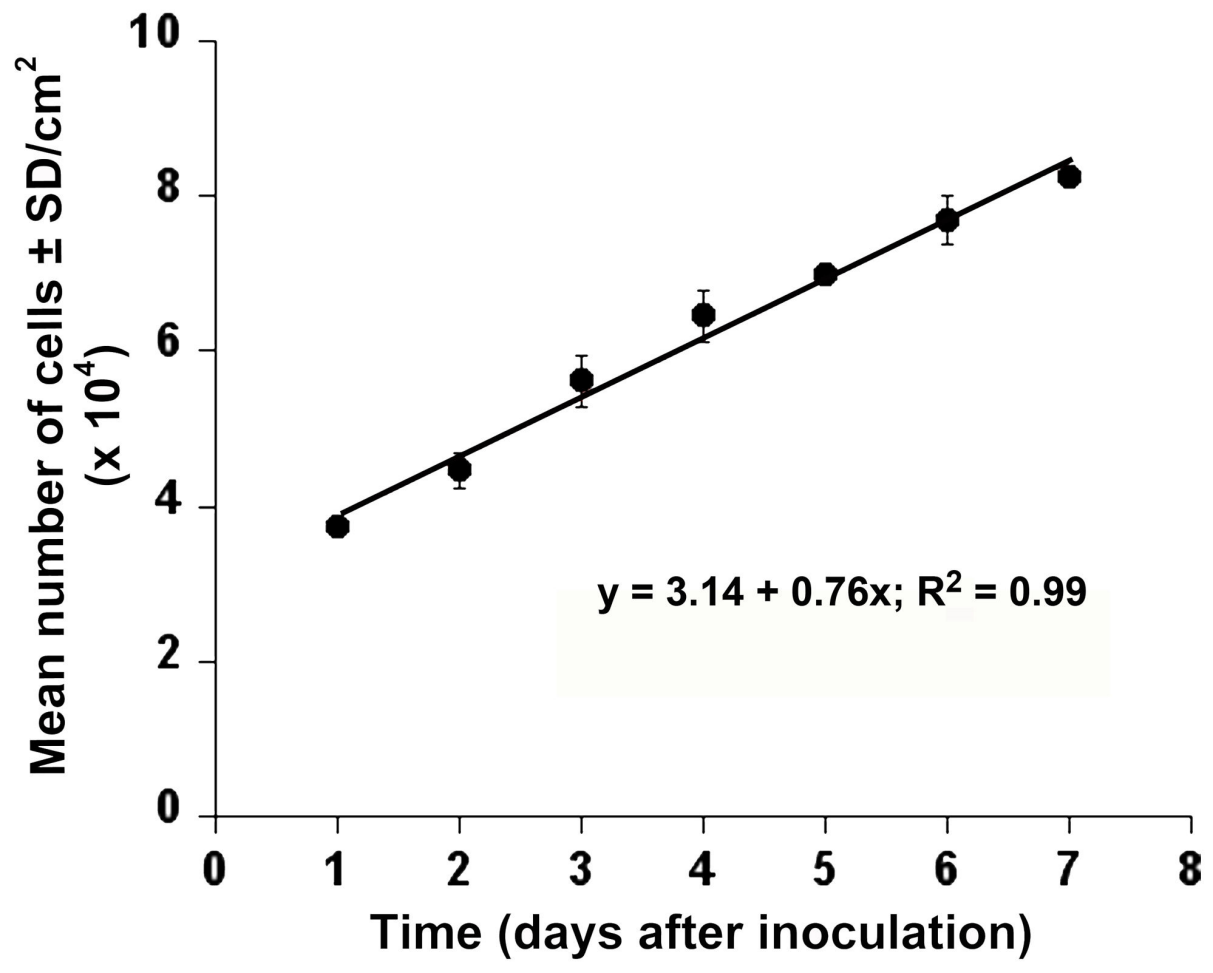
Proliferation of AmE-711 cells *in vitro*. The number of cells was counted from 10 random fields from cultures that had been transferred 12 times. Values are expressed as the mean number of cells per cm^2^ surface area ± SD of 3 replicate cultures.

AmE-711 cells were split at a 1:3 ratio and maintained in the presence of 10% FBS at 32°C. Under these conditions, the population doubling time was estimated to be approximately 4 days (y = 3.14 + 0.76x; r^2^ = 0.99) during the exponential growth phase (Figure 6). To gauge the mitotic index, cells were incubated with 0.125 mM colchicine in hypotonic KCl solution for 40 min according to the method described by Brito and Oldroyd [36]. The frequency of cells in metaphase arrest, or those cells with nuclei that had clearly visible chromosomes, after this pulse of colchicine was 0.8% (40 out of 5,000 spreads examined).

**Figure 7:**
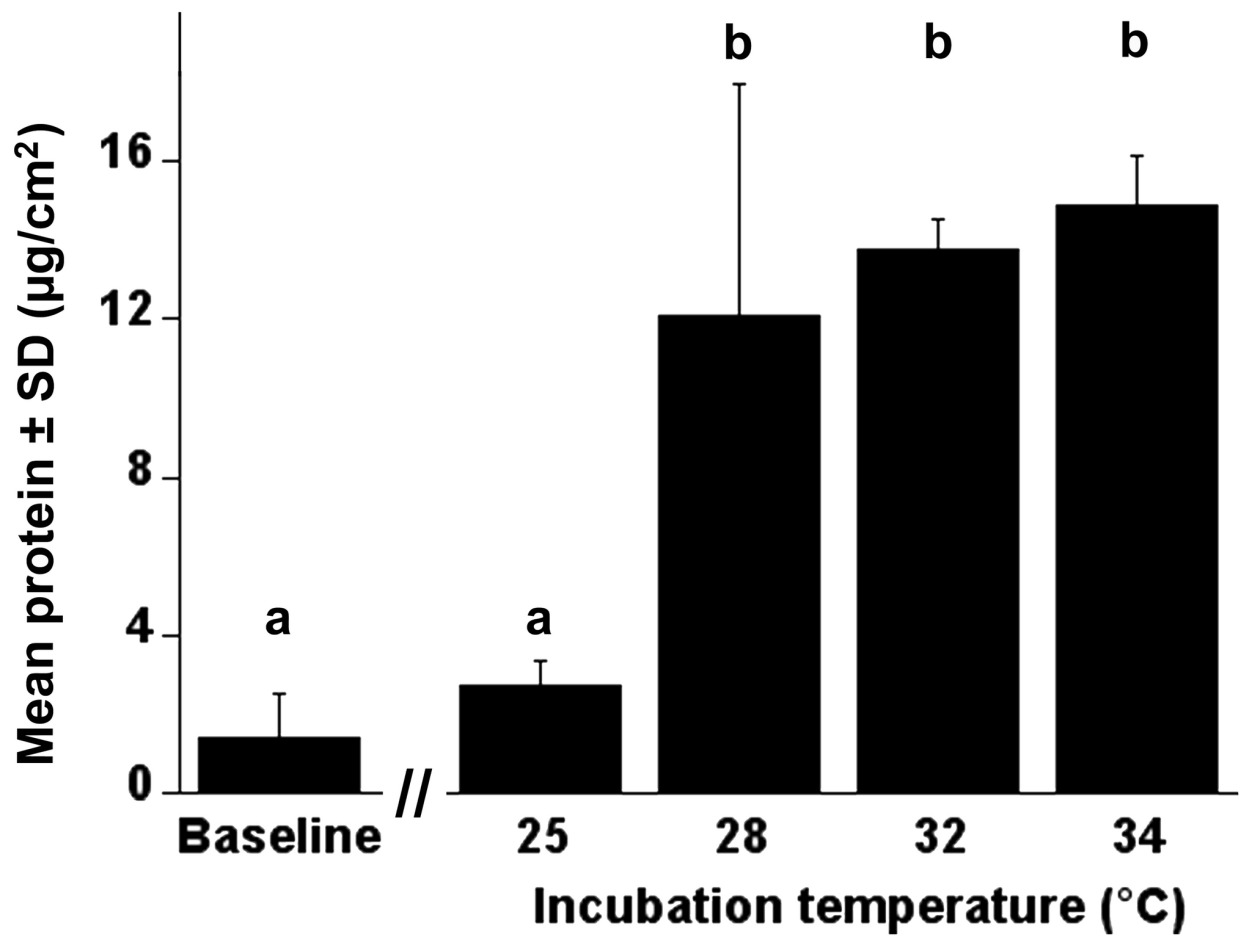
Expression of proteins in AmE-711 cells at different temperatures. The AmE-711 line had been transferred 12 times and incubated for 7 days at different temperatures. Mean total protein per unit surface area of tube substrate (cm^2^) was determined from 3 replicate cultures per temperature using a Bradford assay. Baseline data represent the amount of protein in cells from 3 replicate cultures harvested within 24 h after inoculation. All cultures were run in triplicate and the data were normalized against a reagent blank (0.5 N NaOH) and BSA standard curve. Mean total protein was significantly different between cultures grown at different temperatures (*F*_4,10_=16.00, *p*=0.0002). Columns with different letters are significantly different by Tukey’s HSD (α = 0.05).

A one-way ANOVA was conducted to compare the effect of incubation temperature on total protein expression, which served as a proxy for cell proliferation. There was a statistically significant difference in mean total protein per cm^2^ of tube substrate for cultures incubated at different temperatures (*F*_4,10_=16.00, *p*=0.0002). Interestingly, cultures that had been incubated at 25^°^C for 7 days had protein levels (2.70 ± 0.67 µg/cm^2^) that were significantly lower compared to cultures incubated at 28^°^C (12.08 ± 5.88 µg/cm^2^), 32^°^C (13.76 ± 0.78 µg/cm^2^), and 34^°^C (14.89 ± 1.27 µg/cm^2^) (Figure 7). There was no difference in total protein for cultures incubated at 28, 32, and 34^°^C. Genersch et al. [29] suggest that incubation temperature has no effect on *in vitro* growth of honey bee cells. These authors report no difference in growth between cultures maintained at room temperature (18–25^°^C) and those maintained in the range of 27–31^°^C [29]. Although we saw considerable variability in protein content for replicate cultures at 28^°^C, our finding would suggest that there is a range at which cultures will display greater proliferation.

**Figure 8:**
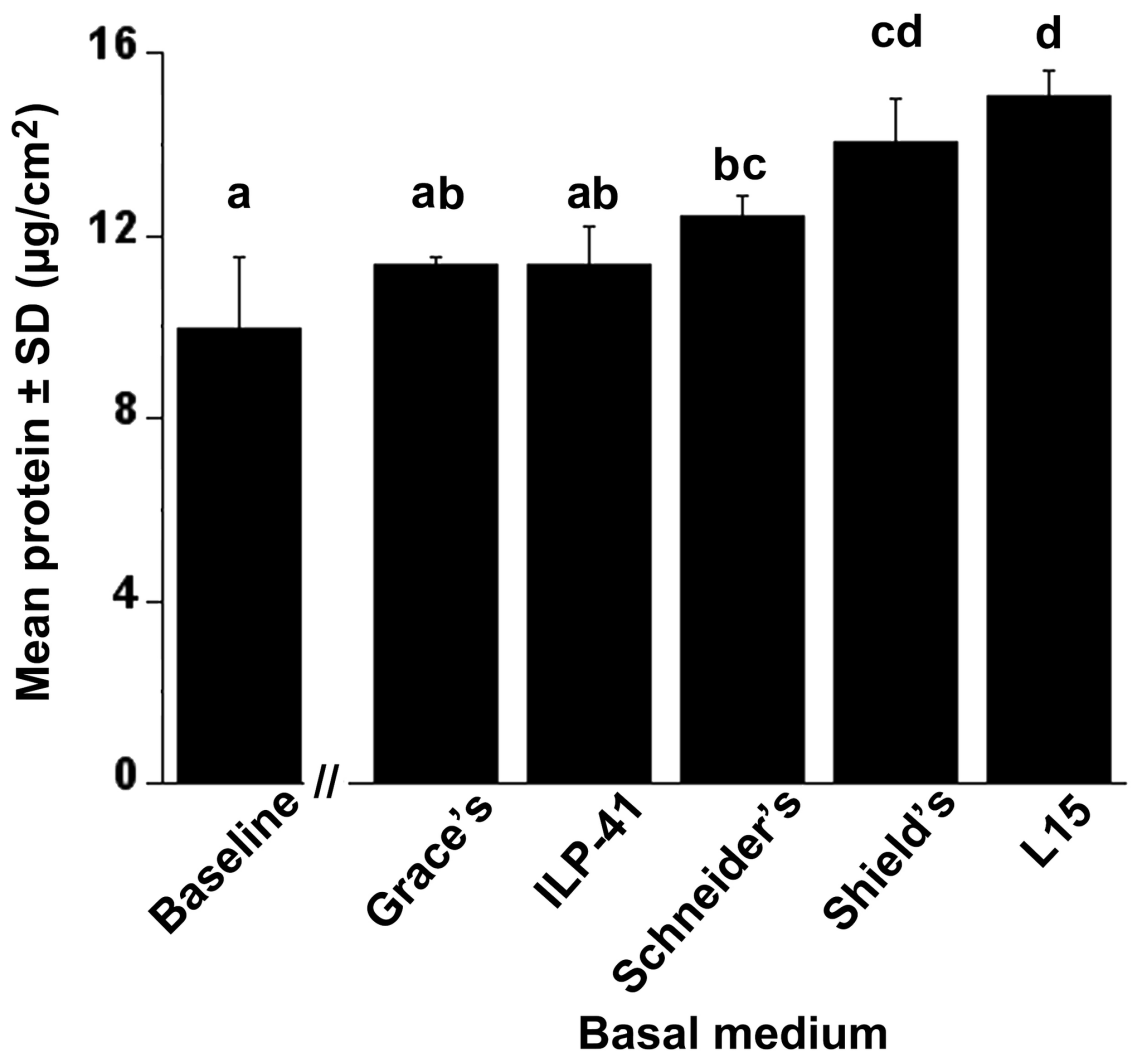
Expression of proteins in AmE-711 cells grown in different media. AmE-711 cells had been transferred 15 times and incubated for 5 days in different commercially available media. Mean total protein per unit surface area of tube substrate (cm^2^) was determined from 3 replicate cultures per base medium using a Bradford assay. Baseline data represent the amount of protein in cells from 3 replicate cultures harvested 48 h after inoculation and before the HB-1 media of the remaining cultures was replaced with a random assignment of the following base media: Grace’s, ILP-41, L15, Schneider’s, and Shield’s. All cultures were run in triplicate and the data were normalized against a reagent blank (0.5 N NaOH) and BSA standard curve. Mean total protein was significantly different between cultures grown in the presence of different base media (*F*_5,12_=13.64, *p*=0.0001). Columns with different letters are significantly different by Tukey’s HSD (α = 0.05).

We tested the adequacy of other commercially available media in addition to L15 on the maintenance of the AmE-711 line and found a statistically significant difference in cell growth based on mean total protein per cm^2^ of tube substrate (*F*_5,12_=13.64, *p*=0.0001) (Figure 8). Proliferation was greater for cultures grown in L15 (i.e., HB-1) (15.04 ± 0.58 µg/cm^2^) compared to Grace’s (11.35 ± 0.16 µg/cm^2^), ILP-41 (11.36 ± 0.85 µg/cm^2^), and Schneider’s (12.40 ± 0.48 µg/cm^2^), but not Shield’s (14.05 ± 0.95 µg/cm^2^). Interestingly, Bergem et al. [12] reported poorer growth outcomes with L15 as a base as opposed to Grace’s, but this could be due to other factors such as differences in supplementation of the complete medium, initial seeding density, or type of cultureware used. We are conducting further experiments to determine if the medium developed by Hunter [19] and others sustain growth similar to what we have observed with L15 and whether the AmE-711 line can adapt to Shield’s as a base.

## Discussion

Cell culture is a remarkable technique, in that populations of cells from tissues explanted from a whole animal or plant can continue to grow when inoculated into a sterile flask containing a physiologically-relevant medium. Cells in primary culture represent a simplified environment that approximates the *in vivo* condition; however, these cells normally undergo senescence resulting from finite proliferation and differentiation capability [37]. Therefore, the time frame to use primary cultures in applications may be limited. Interestingly, some cells in primary cultures can undergo alteration, either naturally through the accumulation of mutations or artificially by transfection. Altered cells have the capability to divide continuously and become confluent within the growth medium. In cultures that have cells that continuously divide, it is possible to transfer some of this material to daughter or subcultures to create lines where the number of cells expands and the lifespan of the culture is prolonged indefinitely (i.e., immortalized).

We have developed an *in vitro* system that allows for long-term maintenance of primary cultures and young cell lines derived from honey bee embryonic tissues. We found that it was not unusual for several of our primary cultures to remain mitotically active for more than a year after they were initiated, and this allowed for the eventual subculture and isolation of a line that has undergone 18 transfers (at the time of submission of this manuscript). From growth cycle and protein expression analyses, we estimate that the AmE-711 line undergoes at least 2.4 doublings between passages. This would equate to a minimum of 43 generations since its isolation, which is in the range of the hypothetical threshold of 40–50 generations that delineates when most mammalian cell lines cease mitotic activity and enter senescence and a few lines become altered with the potential for immortalization [37]. We have taken the necessary steps to ensure a supply of cells through cryopreservation, especially at early passages. We have successfully recovered cells from cryopreservation and these cells are viable and mitotically active. This hallmark in cell line development will allow long-term maintenance of the AmE-711 line and permit the potential distribution of this resource with other laboratories. Furthermore, our low success rate in establishing lines (1 line from ~ 100 primary cultures) and the long period of adaptation (~ 1 year) is not unexpected for insect cell culture [12,38]. However, it alludes to the fact that more work is needed, using the AmE-711 line as a platform, to increase success and shorten the period of adaptation, as well as promote selection of different cell types similar to what is reported for dipteran and some other insect lines [39].

Cell culture is imperative for infection and axenic growth of microbes that are causative agents of disease. Many factors such as growth medium, culture age, and cell type and surface structure limit host range [40]; therefore, future research will need to ascertain the susceptibility of the fibroblast-type cells in the AmE-711 line to specific pathogens. The utility of an *in vitro* culture system comprised of honey bee cells has recently been demonstrated with research aimed at elucidating virulence factors of the bacterium that causes American foulbrood, *Paenibacillus larvae*. Poppinga et al. [41] used pupal midgut cells in primary culture to identify a shape-determining protein of *P. larvae*, whose predicted function permits adhesion to the host cell. Two other pathogens of honey bees, *Nosema* *apis* and *N* *. ceranae* , are obligate intracellular fungi that are widespread in beekeeping operations [42]. *Nosema* *ceranae* is considered an emerging pathogen and debate persists as to whether it is more virulent than *N* *. apis* [43,44], which has been recognized in honey bee colonies since the early 20^th^ century. Remarkably, it has been demonstrated that both species of Microsporidia can infect the heterologous lepidopteran cell line, IPL-LD-65Y, derived from ovarian tissues, which is neither tissue nor host specific for these highly-evolved fungi [45]. This finding broadens the potential use of the AmE-711 line for studying host cellular responses to not only these, but other honey bee pathogens, such as the many viruses to which we have limited knowledge of their infectivity and pathogenicity [46]. Moreover, efforts have been initiated in our laboratory to determine whether AmE-711 cells can be infected with *Nosema* sp. spores.

## Conclusions

Declining honey bee populations in North America and Europe [47] has been the impetus for considerable research efforts aimed at mitigating the many challenges thought to be responsible for this crisis. This decline has resulted in increased inputs (e.g., labor, expenses) by beekeepers to monitor and treat colonies in crisis, as well generates uncertainty in the supply of colonies for commercial pollination or other bee-derived products. In tandem with the recently sequenced genome of *A. mellifera* [48], an *in vitro* system comprised of honey bee cells will facilitate studies in functional genomics and/or target mechanisms of pathogenesis through infection with viruses, for example. Honey bees are also host to a plethora of non-pathogenic, horizontally acquired microbes [49]; a host-derived culture system could cast light on mechanisms of host tolerance and nutritional consequences of harboring these symbionts. In addition, the introduction of foreign gene constructs or knockdown of specific gene targets through RNA interference are attractive applications that would permit questions to be asked about subcellular and molecular changes involved with honey bee development or metabolic responses to pesticides and other xenobiotics.
